# An Investigation of the Effects of the Acceptance and Commitment Therapy in Groups on the Cognitive Strategies of Emotion Regulation and Self-Control in Coronary Heart Disease Patients

**DOI:** 10.25122/jml-2019-0035

**Published:** 2019

**Authors:** Hossein Sheibani, Khadijeh Aerab Sheibani, Nazanin Nezhad Amreei, Mojgan Javedani Masrour

**Affiliations:** 1. Assistant Professor of Cardiology, Clinical Research Development Unit, Imam Hossein Hospital, Shahroud University of Medical Sciences, Shahroud, Iran.; 2. Assistant Professor, Department of Psychology, Payame Noor University, Tehran, Iran; 3. Assistant Professoe, Master’s Degree in Psychology, Department of Psychology, Payame Noor University, South Khorasan, Birjand, Iran; 4. Assistant Professor, Research & Clinical of Gynecology and Fertility, Shahid Akbar-Abadi Hospital, Iran University of Medical Science, Tehran, Iran

**Keywords:** Acceptance and commitment therapy, cognitive strategies of emotion regulation, self-control, cardiovascular patients

## Abstract

Coronary heart diseases are amongst the most common and severe diseases; also, the prevalence and emergence of these diseases are largely affected by psychological and social factors. The present study was conducted in order to investigate the effects of acceptance and commitment therapy in groups on the cognitive strategies of emotion regulation and self-control in patients with cardiovascular diseases.

The present work is a quasi-experimental research with a pretest-posttest design and a control group.

The statistical population of this study included all patients with coronary heart disease aged 35-55 years who referred to hospitals, health centers, and cardiovascular clinics of Isfahan in the spring of 2018. Among these patients, 30 patients were selected based on the inclusion criteria of the study using a convenience sampling method. They were then divided randomly into two experimental and control groups, with 15 participants in each group. The patients in the experimental group participated in eight 60-minute sessions of the Hayes’ Acceptance and Commitment Therapy (ACT) (2002). However, no intervention was applied to the control group. The participants responded to the research questionnaires in two phases. The questionnaires included the Garnefski’s Cognitive Emotion Regulation Questionnaire (CERQ) (2001) and the Tangji’s self-control questionnaire (2004). The research data were analyzed using covariance analysis in SPSS 24.

The results of the covariance analysis indicated that the group-based acceptance and commitment therapy exerted a significant effect on the total score of the two strategies, i.e., the positive strategy of the cognitive regulation of emotions and the negative strategy of the cognitive regulation of emotions (p<0.05). However, no significant difference was observed in terms of self-control (p> 0.05).

The findings of the present study showed that the treatment intervention resulted in increased use of acceptance strategies, positive re-focus, and re-focus, as well as reduced use of rumination and other-blaming strategies in cardiovascular patients in Isfahan.

## Introduction

Nowadays, cardiovascular diseases are considered the most common and severe type of diseases in developed countries. Also, cardiovascular diseases are regarded as the leading cause of mortality and lost years of life. In Iran, about 35% of the entire mortality rate is due to cardiovascular diseases. Since cardiovascular and heart coronary diseases are regarded as psychosomatic, a combination of biological and psychological factors is involved in their emergence [1]. There is a large body of evidence in psychological and social studies on coronary heart diseases and their risk factors concerning the effects of the intervention of social environments and individuals’ coping styles on their health [2]. In fact, coronary heart diseases not only affect patients’ happiness and welfare but also influence their social interactions, life patterns, jobs, income levels, and welfare in general [3]. Moreover, some factors, such as anger, aggressiveness, emotional suppression, and failure to control one’s emotions, depression, and pessimism, are positively correlated to cardiovascular diseases [2].

Emotions are among the factors which are likely to exert a significant effect on the occurrence of psychosomatic diseases. Emotions are an indispensable part of personal and social adjustment, without which individuals’ life will have no meaning, structure, richness, happiness, and interaction with others. Besides, emotions exert a significant effect on the various aspects of life. Emotions are also useful in adapting to stressful events and life changes [4]. Most studies conducted on emotions try to regulate their effects on individuals’ behavior and cognition. Emotion regulation is regarded as a procedure through which individuals adjust a form of emotions they experience at the moment and the way they regulate the manner of experiencing and expressing their own emotions [5]. Emotional regulation requires the application of behavioral and cognitive strategies for changing the duration or intensity of emotions. It is proven that individuals employ different emotional regulation strategies in dealing with stressful events to improve or modify their emotional experiences [6]. One of the most common methods of emotional regulation is the employing of cognitive strategies. Cognition or cognitive procedures help individuals regulate their emotions and feelings to prevent emotions from conquering them. The cognitive regulation of emotions refers to the cognitive method of managing and manipulating the information of the emotion elicitor [7]. Numerous personal factors, including the character and history of mental and environmental health (the family and job environments), affect emotion regulation and control. The emotion regulation process seems to be disrupted in acute and chronic diseases [8], with adaptive approaches replaced by maladaptive strategies. Thus, applying psychological interventions seems to be necessary in such cases.

Among the primary factors preventing the incidence of coronary heart diseases could be controlling or failing to control behaviors and emotions in different situations of life. Self-control was developed by Schneider in 1974, which refers to the individual’s flexibility or stability in different situations [9]. Moreover, this concept reflects individuals’ differences in desires and takes a definite form in managing feelings and emotions [10]. Bandura’s Social Learning Theory implies that self-control can be learned and taught [11]. Schneider et al. stated that people could be divided into two classes, i.e., individuals with high self-control and those with low self-control [12]. Either of these two groups has its features. Some individuals are sensitive to social situations and adapt themselves to the status quo. These individuals are the ones with high self-control.

In contrast, there are individuals with low self-control, who do not express their ideas and feelings unless they try to manage or organize them in accordance with their situations [13]. Individuals with internal self-control potentials prepare themselves in dealing with events and believe that their actions and behaviors, rather than accidental and external factors, lead to events. One of the main advantages of self-control is the regulating of emotions and controlling of arousal levels to maximize proper actions and prevent destructive reactions to imposed stimulations and pressures. Any failure in self-control will result in unsuccessful social adjustment as well as physical and mental health [14].

Nowadays, therapists are dealing with the third generation of treatments. They have included acceptance-based models, mindfulness-based cognitive therapy (MBCT), and the acceptance therapy (ACT) [15]. In these therapies, instead of changing cognitions, it is attempted to promote individuals’ psychological relationship with their thoughts and feelings. In doing so, these therapies aim to help patients have a more valuable and satisfactory life by increasing their psychological flexibility rather than focusing on cognitive restructuring [16]. Among the third wave of psychological approaches, acceptance and commitment therapy enjoys a high level of effectiveness in controlling individuals’ attitudes and perceptions against stressful events [17].

Acceptance and commitment therapy is a behavioral therapy that employs mindfulness skills, acceptance, and cognitive diffusion to promote psychological flexibility. Acceptance and commitment therapy results from patients’ capability of creating a relationship with their current experiences based on the things which are possible for them at that moment [18]. In fact, acceptance and commitment therapy is a context-oriented approach that challenges patients in a way that they will accept their thoughts and feelings and commit themselves to make the required changes [19]. In the acceptance and commitment theory, it is tried to make individuals improve their living conditions without avoiding disturbing thoughts and feelings by providing examples and comparisons. Research indicates that the acceptance and commitment therapy has been applied to a wide range of psychological issues, including anger, anxiety, depression, obsession, social phobia, drug abuse, and psychoses [16]. The positive results of improved disorders and the reduced severity of symptoms have been reported in the studies conducted using this approach.

In a study conducted by Baseri and Bozorgi [17], the authors reported that the applying of the group-based acceptance and commitment therapy increased the cognitive regulation of emotions significantly and reduced alexithymia in patients with type 2 diabetes. In another study, Abbasi, Khazan, Pirani, and Ghasemi [18] reported that the controlling of the effects of the pretest and the applying of the acceptance and commitment therapy in the experimental group resulted in the improvement of self-control, self-motivation, emotional self-regulation, and the cognitive-emotional function. In their study, Boostani, Ezadikhah, and Sadeghi [19] discovered that the intervention group experienced a significantly more reduction in the difficulty with emotional regulation after receiving the required intervention than the control group. Besides, the level of tension tolerance improved significantly in the intervention group. Spatola et al. [20] also discovered that acceptance and commitment therapy was effective in reducing patients’ tension and distress. In their study, Ryan et. al and others stated that acceptance and commitment could help regulate adults’ negative behavioral patterns and automatic thoughts, resulting in the regulation of positive social behaviors related to health [21-23].

Since coronary heart diseases create numerous problems for individuals and psychological factors are the better indicators of disease adjustment, the present study was conducted in order to answer the following question: ‘does the group-based acceptance and commitment therapy affect the cognitive strategies of emotion regulation and self-control in coronary heart disease patients of Isfahan?’

## Material and Methods

The present work is a quasi-experimental research with a pretest-posttest design and a control group. In this study, the aim of the independent variable is based on the acceptance and commitment and purpose of dependent variables of the cognitive strategies of regulation of emotion and control itself. The overall aspect of the research design is presented in Table 1:

**Table 1:** The general aspect of the research design.

Row	Groups	Groups of random	Pretest	Asignment of the independent variable	Posttest
**1**	Acceptance - based treatment and commitment	ER	T1	X	T2
**2**	Control group	ER	T1	-	T2

As deduced from above, both groups were measured in two phases. Before and after the execution of the independent variable, the witness group was not subjected to any intervention.

The statistical population of this study includes all coronary heart patients aged 35-55 years, who referred to hospitals, health centers, and cardiovascular clinics of Isfahan in the spring of 2018. All participants in this research have no history of psychiatric disorders, and there were no diseases associated with coronary artery disease [24].

Among these patients, 30 patients were selected based on the inclusion criteria of the study using a convenience sampling method. They were then divided randomly into two experimental and control groups, with 15 participants in each group. Since the present research project has the study design described by Cook et al. (quasi-experimental), in the semi-experimental research, the selection of 15 sample sizes is sufficient for each group [24, 25].

The two groups were then asked to complete the questionnaire. The inclusion criteria of the present study included (1) the diagnosis of suffering from coronary heart diseases by a cardiologist, (2) being within the age range of 35-55 years, (3) having the minimum basic literacy level of reading and writing as well as understanding the questions of the questionnaire, (4) being fully conscious, able to talk, and physically and mentally prepared to answer the questions, and (5) giving informed written consent to participate in the study. The exclusion criteria were (1) suffering from a severe physical disease and having physical disabilities, (2) suffering from acute psychiatric disorders, (3) missing more than two sessions of the ‘acceptance and commitment therapy’ training course, and (4) being unwilling to continue cooperation.

### Statistical analysis

SPSS 24 was used for the statistical analysis of the research data. The indicators of descriptive statistics, including the mean and the standard deviation, were applied to describe the data, and covariance analysis was utilized for inferential statistics. The data collection tools of the present study included the Garnefski’s Cognitive Emotion Regulation Questionnaire (CERQ) (2001) and the Tangji’s self-control questionnaire (2004).

1. Cognitive Emotion Regulation Questionnaire: The cognitive Emotion Regulation Questionnaire is a 36-item self-report. Garnefski, Kraaij, and Spin-Hoven developed this questionnaire in 2001, and it is applied to identify cognitive coping strategies after an unpleasant experience. This questionnaire has nine components, five of which (acceptance, refocus on planning, positive refocusing, positive reappraisal, and putting into perspective) are the positive strategies of emotion regulation and four of which (self-blame, blaming others, rumination, and catastrophizing) indicate the negative strategies of emotion regulation. The answers to the questions were evaluated on a five-scale continuum (always, often, regularly, sometimes, and never). According to the designers of the questionnaire, the validity values of this questionnaire (by applying Cronbach’s alpha) have been measured to be 91.0 for positive strategies, 87.0 for negative strategies, and 93.0 for the entire questionnaire. Using Cronbach’s alpha, the validity and reliability of the questionnaire in the Iranian population have been measured to be 70.0 for the entire questionnaire [26].
2. Tangji’s Self-control Questionnaire: This questionnaire was developed by Tongji et al. in 2004, and it consists of two subscales, i.e., preventing or inhibiting self-control and primary self-control. It has 36 questions, with its main aim being to measure individuals’ control on themselves. The answers were placed on a five-point Likert scale (ranging from 5=always true about me to 1=never true about me), with higher scores indicating a higher level of control on one’s behavior. In another study, De Ridder et al. [23] investigated the differences among these subscales. They analyzed the internal consistency of this questionnaire by the alpha report for both subscales, with the Cronbach’s alpha being 86.0 and 68.0 for inhibiting self-control and primary self-control. In the study conducted by Atlasi, Narimani, and Musa Zadeh [27], the correlation among the subscales was measured to be 72.0.

For the purpose of this study, 30 individuals who met the inclusion criteria were selected. They were then divided randomly into two experimental and control groups, with 15 participants in each group. Next, both groups were asked to fill out the research questionnaires carefully. After doing the pretest, the participants of the experimental group underwent a medical intervention of eight 60-minute sessions by providing them with the Hayes’ Acceptance and Commitment Therapy (ACT) package. In contrast, no intervention was applied to the control group. For the posttest, both groups were asked to fill out the questionnaire (cognitive emotion regulation and self-control) once more. The content of the sessions has been provided in Table 2.

**Table 2:** Sessions described in detail as well as the Hayes’ Group-based Acceptance and Commitment Therapy (2002).

Sessions	The session’s contents
**The 1st session**	Introducing the members, providing an overview of the structure of the sessions and the main rules of the group, the number of sessions, and the duration of each session, expressing the expectations from the sessions, knowing the participants, and administering the pretest.
**The 2nd session**	Expressing the positive and negative thoughts and feelings of the members about the disease as well as physical and mental harms arising from the disease, providing a general evaluation, expressing the type of the therapeutic communication using the metaphor of the two mountains and mountain climbers, introducing the concepts of mind and language according to ACT, assigning homework for writing the daily experiences of mind products.
**The 3rd session**	Reviewing homework in an attempt to provide a better definition of the mind, mind products, as well as control and prevention strategies by providing the metaphor of the pit and the shovel, and assigning homework for a better understanding of shovels and pits.
**The 4th session**	Reviewing homework, assessing creative hopelessness, investigating pits and shovels as well as control strategies, investigating and evaluating control and prevention behaviors, providing a table for the investigation of the consequences of control strategies, and specifying the inefficiency of reactions to inefficient thoughts.
**The 5th session**	Reviewing homework and reactions, discussing the previous session, elaborating self-control, confirming the uselessness of the control on internal events (thoughts and feelings), encountering experiences with a harmless nature.
**The 6th session**	Teaching acceptance, diffusion, or willingness using the guest-host metaphor, teaching the members making use of the bus metaphor for the concepts of diffusion and fusion, providing an exercise for members in terms of the lack of fusion using one’s mental products and achieving goals and values through diffusions from one’s mind, teaching pleasant and unpleasant pains, and providing a table for recording pleasant and unpleasant pains.
**The 7th session**	Specifying values and targets making use of the bus metaphor, providing the members with a table of values, helping the members identify values and targets on their path of values in the presence of the researcher and other members, identifying oneself as the context using the chessboard metaphor, and teaching the method of reaching oneself using values, targets, and diffusion.
**The 8th session**	Describing committed actions and behaviors, drawing a conclusion to prevent the members from returning to their futile actions, presenting the ACT psychological flexibility six processes, presenting the ACT psychopathology six processes, and administering the posttest.

## Results

The mean age of the patients participating in the present study was 53.46 and 33.50 in the control and experimental groups, respectively. In addition, the mean age of the statistical population was 43.48. Due to the particular case of this research, patients were selected as available if they were from the adult age group and were randomly tested in two groups of test and control groups. The age difference between the two groups of experimentation and control is not fundamental.

Among the patients, 26.7% of them were suffering from the disease for one year or less. Also, 40% of the patients were suffering from the disease for 1 to 4 years, and 33.3% of them were suffering from the disease for more than 4 years. Besides, 40% of the participants in this study had a family history of cardiovascular diseases. However, 60% of the participants had no family history of cardiovascular diseases. Concerning the educational level, 33.3% of patients had a high school diploma, 43.4 % had a diploma and associate degree, and 23.3 % had a bachelor’s degree and master’s degree. In terms of family history, 40 % of people participating in research had a family history of cardiovascular disease, while 60% did not have a family history.

To examine the normality of score distribution, the Kolmogorov–Smirnov test, the Shapiro–Wilk test, and histograms were employed. As a result, the normality of score distribution was confirmed for all three variables (P<0.05). The results of Levene’s test for the positive strategies of emotion regulation (F=0.054, p=4.04), the negative strategies of emotion regulation (F=0.38, p=0.77), and self-control (F=0.61, p=0.26) confirmed the assumption of variance equality in both experimental and control groups (P>0.05). Besides, given the Box’s M statistic (67.11) and the significance level obtained (0.11) in the Box’s M test, the equality assumption of variance-covariance matrices was confirmed (P>0.05).

The results of the Wilks’ lambda test (Table 3) indicated that the acceptance and commitment therapy was significant at least for one of the variables of the positive strategies of cognitive emotion regulation, the negative strategies of cognitive emotion regulation, and self-control (P<0.05). The effect size (eta-squared) indicated that 33% of the variance of the dependent variables was measured for the independent variable. In addition, the statistical power of the test (0.75) indicated the adequacy of the sample size.

**Table 3:** The mean and the standard deviation of the control and experimental groups based on the measurement phase.

Scale	Phase	Control		Experimental	
		Mean	Standard deviation	Mean	Standard deviation
**Positive strategy of emotion regulation**	Pretest	56.80	51.80	54.06	61.80
	Posttest	8.11	7.89	7.26	10.75
**Negative strategy of emotion regulation**	Pretest	59.53	55.20	58.33	50.20
	Posttest	9.07	7.80	9.27	83.7
**Self-control**	Pretest	87.86	8633	74.13	88.40
	posttest	24.92	18.94	2050	23.35

Table 4 demonstrates the results of inter-group and intra-group variance analyses to determine the effects of the acceptance and commitment therapy on the three variables of the study in two measurement phases.

**Table 4:** Wilks’ lambda test for examining mean differences.

**Values**	**F**	**df1**	**df2**	**Significance level**	**Effect size**	**The statistical power of the test**
0.666	85.3	3	23	0.023	0.33	0.75

The results of Table 5 indicate that among the three variables introduced, the difference in the mean scores of the participants in terms of both positive strategies of cognitive emotion regulation (F=10.25) and negative strategies of cognitive emotion regulation (F=4.47) was significant between the experimental and control groups, after eliminating the effects of the pretest (P<0.05). However, such a significant difference was not observed for the variable of self-control (P>0.05). The Eta-squared value of the effect size indicated that 29% of the differences of the positive strategies of emotion regulation and 15% of the differences of the negative strategies of emotion regulation in the posttest were related to the differences between the groups, which were measured for the independent variable of the acceptance and commitment therapy.

**Table 5:** The results of covariance analysis for the scores of the positive strategies of cognitive emotion regulation, the negative strategies of cognitive emotion regulation, and self-control.

Variable	Resource change	Sum of squares	df	Mean squares	F	Significance level	Eta-squared	Statistical power of the test
**Positive strategies of emotion regulation**	Pretest	318.5	1	318.05	3.93	0.058	0.13	0.48
	Group	829.18	1	829.18	10.25	0.004	0.29	0.86
**Negative strategies of emotion regulation**	Pretest	7.84	1	7.84	0.010	0.701	0.01	0.06
	Group	232.67	1	232.67	4.47	0.045	0.15	0.53
**Self-control**	Pretest	3449.2	1	3449.2	28.45	0.001	0.53	0.99
	Group	97.18	1	97.18	0.203	0.656	0.08	0.07

## Discussions

The present study examined the effects of acceptance and commitment therapy on the cognitive strategies of emotion regulation and self-control in cardiovascular patients. According to the results, the group-based acceptance and commitment therapy increased individuals’ skill in employing the positive strategies of emotion regulation and decreased the employing of the negative strategies of emotion regulation in patients with coronary heart disorders.

Although no study was found to be precisely similar to the current one, the results of the present study were in line with those conducted by Baseri and Bozorgi [17], Abbasi et al. [18], Boostani et al. [19], and Spatola et al. [20]. However, the results of the current study were not consistent with those conducted by Ryan and Deci [21]. In explaining the effectiveness of the acceptance and commitment therapy in emotion regulation strategies, researchers have referred to the significant effects of the maladaptive patterns of emotion regulation on individuals suffering from depression, anxiety, and psychosomatic diseases. Based on these theories, depression results from individuals’ failure to regulate their emotions adaptively and properly. In fact, the main hypothesis is that individuals’ capability for regulating their emotions in a desirable manner and their toleration of undesirable emotions are the main factors that influence mental health [27].

The acceptance and commitment approach emphasizes individuals’ full vigilance and disease acceptance [28]. Without making any attempts to control the thoughts about the disease, individuals let the underlying or disease-related thoughts and emotions enter their minds. When such experiences, i.e., thoughts and emotions, are undergone with acceptance, the hardest problems seem to be less threatening and more tolerable, with ineffective controlling actions being reduced by a significant level [27]. Moreover, acceptance and commitment therapy has managed to affect clients’ emotion regulation by focusing on flexibility. Relaxation and mindfulness are emphasized in acceptance and commitment therapy exercises. They exert significant effects on individuals’ peace of mind and reduce stress as well as automatic negative thoughts.

In addition, acceptance and commitment call for the active and patient acceptance of hard feelings so that individuals’ reactivity, as well as improper fears and judgments, are reduced to a great extent. Research has implied that such feelings increase interpersonal discomforts and tensions and pave the way for empirical avoidance. It seems that using this method, patients learn how to put an end to their discomforts concerning their anger at the disease, blaming others for their disease, and ruminating about negative thoughts concerning their disease. Instead, individuals try to get involved in activities that make them get closer to their life goals and values. In doing so, individuals will exert better control over their actions. Furthermore, the insignificance of the effects of the intervention on self-control could be due to the patients’ mental and personality features. For instance, individuals with ‘type A personality’ attempt to control all life aspects, even the uncontrollable ones; also, they tend to get excited and emotional in this regard.

Moreover, they are very likely to place themselves in stressful conditions and choose the most stressful and most laborious solutions in stressful conditions. Failure to manage behaviors and personal feelings, as well as the lack of resistance against short-term temptations, will result in stress, hostility, unhappiness, aggressiveness, and exacerbated heart problems.

Just like other similar studies, the present one has its setbacks and limitations. The first limitation of the present study was that due to the time limitation, there was no time for conducting the second follow-up test and measuring the consistency of the effects of the intervention based on the acceptance and commitment therapy at different intervals. Moreover, given the nature and the inclusion criteria of the present study, the random selection of the samples was not possible; thus, the convenience sampling method was employed, with the participants divided randomly into experimental and control groups. Therefore, it is recommended that follow-up evaluations be carried out to assess the effect changes of the interventions over time. The consistency of the results of this study is implemented and can be presented in a separate article concerning the measurement of stability effect of the intervention of group care based on acceptance and commitment in patients with coronary heart complications. The quality of life and eating habits, smoking, and alcohol consumption are variables that can be considered separately in the form of health care programs.

Studies that are investigating other psychological and biological variables are carried out, and the results will be presented to complete the results of this research.

Furthermore, it is suggested that the effects of family conditions, occupational conditions, and social factors be examined, being key factors in heart diseases.

## Conclusion

The results of the present study showed that the treatment intervention resulted in increased use of acceptance strategies and positive refocus, as well as reduced use of rumination and other-blaming strategies in cardiovascular patients from Isfahan. According to the results, the group-based acceptance and commitment therapy increased individuals’ skill in employing the positive strategies of emotion regulation and decreased the employing of the negative strategies of emotion regulation in patients with coronary heart disorders. Although no study was found to be precisely similar to the current one, the results of the present study were in line with those conducted by Baseri and Bozorgi [17], Abbasi et al. [18], Boostani et al. [19], and Spatola et al. [20]. However, the results of the current study were not consistent with the study conducted by Ryan and colleagues [21].

## Conflict of Interest

The authors confirm that there are no conflicts of interest.
